# Airway transcriptome networks for ozone and PM_2.5_ exposure reveal distinct key drivers for children with asthma

**DOI:** 10.1186/s13073-025-01570-1

**Published:** 2025-12-22

**Authors:** Yoojin Chun, Haritz Irizar, Lingdi Zhang, Kyle Reed, Galina Grishina, Alexander Grishin, Alfin Vicencio, Supinda Bunyavanich

**Affiliations:** 1 Department of Genetics and Genomic Sciences, Icahn School of Medicine at Mount Sinai, New York, NY USA; 2 Center for Biostatistics, Icahn School of Medicine at Mount Sinai, Population Health Science and Policy, 1425 Madison Avenue, # 1498, New York, NY 10029 USA; 3 Division of Allergy & Immunology, Department of Pediatrics, Icahn School of Medicine at Mount Sinai, New York, NY USA; 4 Division of Pulmonology, Department of Pediatrics, Icahn School of Medicine at Mount Sinai, New York, NY USA; 5 Department of Pediatrics, Hackensack Meridian School of Medicine, NJ Nutley, USA

**Keywords:** Air pollution, Asthma, Ozone, PM_2.5_, Transcriptome, Airway, Nasal, Bronchial, Adaptive immune, DNA repair

## Abstract

**Background:**

Air pollution disproportionately affects individuals with asthma, triggering asthma exacerbations and morbidity. We hypothesized that children with and without asthma have distinct airway transcriptome networks associated with ozone and particulate matter ≤ 2.5 µm (PM_2.5_) exposure.

**Methods:**

We recruited children from the New York metropolitan area, mapped their air pollutant exposures, and collected nasal and bronchial samples for transcriptome and cellular profiling. We used causal network construction and key driver analyses to build airway transcriptome networks for ozone and PM_2.5_ exposure.

**Results:**

The cohort included 307 children, including 167 (54.4%) with asthma and 140 (45.6%) without asthma. The mean age was 13.4 years (SD 4.4 years). Among children with asthma, the airway causal network and its key drivers represented pro-inflammatory adaptive immune processes. Six key driver transcripts for ozone (*CLC*, *CPA3*, *FGL2*, *LGALS12, IL7R, HRH4*) and three key driver transcripts for PM_2.5_ (*TNFRSF10C, FGL2, EVI2B*) were identified. Nasal expression of *CLC* and *CPA3* positively correlated with respective bronchial expression and bronchial eosinophil abundance. Bronchial expression of *TNFRSF10C* positively correlated with bronchial neutrophil abundance. In striking contrast, among healthy children, key drivers for the ozone network were enriched for DNA repair and immune regulation.

**Conclusion:**

Children with and without asthma have very distinct causal networks and key drivers for ozone and PM_2.5_ exposure. The identified key drivers represent high-yield targets for intervention and treatment of pollutant-exacerbated asthma.

**Supplementary Information:**

The online version contains supplementary material available at 10.1186/s13073-025-01570-1.

## Background

Asthma is a common chronic respiratory disease characterized by shortness of breath, wheezing, cough, and chest tightness. Air pollution has a globally recognized impact on asthma, and many studies have investigated the effects of air pollution on the airway and asthma outcomes [1–15].

Among air pollutants that are commonly monitored, ozone and PM_2.5_ (particulate matter with an aerodynamic diameter ≤ 2.5 µm) have been the focus of much attention, with a large body of evidence supporting that they are associated with asthma morbidity [16–21]. Short-term exposure to ground-level ozone triggers asthma exacerbations, asthma-related emergency department visits, hospitalization, and even death [16–21]. Based on large multi-city epidemiologic studies, exposure to ozone is a recognized risk factor for new-onset asthma, asthma exacerbation, emergency department visits, and hospitalization for asthma [22]. PM_2.5_ can penetrate deep into the lower airway given its small size, triggering inflammation and oxidative stress associated with reduced lung function, especially in children with asthma [1]. PM_2.5_ exposure during early childhood is associated with the development of childhood asthma, with higher risks among families with lower socioeconomic status in densely populated communities [2]. The US Environmental Protection Agency (EPA)’s integrated science assessment weighed the body of experimental evidence, epidemiologic data, and animal toxicology studies, concluding that a “causal relationship” for ozone and “likely causal relationship” for PM_2.5_ exist between short-term exposure to these air pollutants and respiratory outcomes [22, 23]. Ozone and PM_2.5_ have become of increasing concern with more frequent wildfires and longer wildfire seasons in many places around the world, as wildfires significantly increase ozone and PM_2.5_ levels [24–31].

**Fig. 1:**
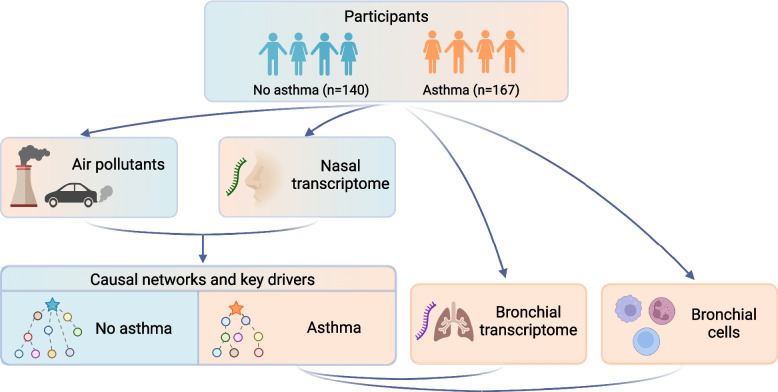
Overview of the study and analytic flow. 307 participants, including 167 children with asthma and 140 children without asthma, were recruited for this study. Air pollutant levels for ozone and PM_2.5_ for participants’ home zip codes were obtained from daily air quality data measured by the U.S. EPA [33]. All participants provided nasal samples and 44 of the asthmatic children who underwent clinically-indicated bronchoscopy additionally provided bronchial samples. Probabilistic causal (Bayesian) network construction and key driver analyses were performed to identify key drivers of airway transcriptional networks for ozone and PM_2.5_ exposure in children with and without asthma. Correlation analyses between the identified key drivers, bronchial transcript expression, and bronchial alveolar lavage cell counts were also performed in children with asthma and bronchoscopic data.

Motivated by this prior work, we hypothesized that children with and without asthma have different airway transcriptome networks associated with ozone and PM_2.5_ exposure, and that probabilistic causal network construction could reveal key drivers that exert causal influence on asthma-associated airway transcriptional programs and cellular profiles. With many prior studies of air pollution exposure and childhood asthma focused on undirected associations and/or a single anatomic site such as the nasal airway given the difficulty of obtaining bronchoscopic samples, particularly in children, we designed this study (Fig. 1) to examine both the upper and lower airways and with causal analyses to identify key drivers predicted to exert causal effects on airway transcriptome networks linked to ozone, PM_2.5_ exposure, and childhood asthma.

## Methods

### Study cohort

The study cohort of 307 participants age 4–26 years was recruited from pediatric clinics in the Mount Sinai Health System in New York, NY, USA and included 167 participants with asthma and 140 without asthma. The study was approved by the Mount Sinai Institutional Review Board. Parents of children provided written informed consent, and assent was also obtained from participants. Children with asthma had physician-diagnosed persistent asthma based on National Asthma Education and Prevention Program (NAEPP): Third Expert Panel on the Diagnosis and Management of Asthma (EPR3) guidelines [32]. In addition to physician-diagnosed persistent asthma, inclusion criteria for participants with asthma included demonstration of airway hyperresponsiveness by pre- and post-bronchodilator spirometry (i.e. increase in FEV1 of > 200 ml and ≥ 12% from baseline after albuterol) or positive methacholine challenge (i.e. FEV1 PC20 ≤ 12.5 mg/ml). Children without asthma were required to have no personal or family history of asthma and demonstrate pre- and post-bronchodilator spirometry within normal limits. Exclusion criteria for both groups included history of non-asthma pulmonary disease, immune disorder, sinus disease, bleeding disorder, or anticoagulant or aspirin use.

### Air pollutant data

Participants’ home zip codes were used to obtain levels of criteria air pollutants including ozone (O_3_), particulate matter (PM), carbon monoxide (CO), nitrogen dioxide (NO_2_), and sulfur dioxide (SO_2_) from the United States Environmental Protection Agency (EPA) [33]. Euclidean distance was used to identify the closest EPA monitoring site for each participant’s home zip code. Mean daily air quality statistics were used for the closest monitoring site for the study visit date.

### Sample collection and transcriptome profiling of airway brushings

Participants were off all medications in the four weeks prior to sampling except for those with severe asthma where withholding asthma medications was not possible. All participants underwent nasal brushing with a sterile cytology brush (Histobrush, Puritan Medical Products, Guilford, ME), and bronchial brushing with a sterile bronchoscopy brush (Cellebrity cytology brush, Boston Scientific, Marlborough, MA) was performed for 44 participants with asthma who underwent clinically indicated bronchoscopy for refractory asthma. No bronchial samples could be collected from the participants without asthma given bronchoscopy is an invasive procedure that is not indicated for healthy individuals. Brushes were immediately placed in RNALater (Thermo Fisher Scientific, Fair Lawn, NJ). RNA extraction was performed using the Qiagen RNeasy MiniElute Cleanup Kit (Valencia, CA). Sample yield and quality were assessed using 2100 Bioanalyzer (Agilent Technologies, Santa Clara, CA) and Qubit fluorometry (Thermo Fisher Scientific, Grand Island, NY). The RNA sequencing library was prepared with the standard TruSeq RNA Sample Prep Kit v2 protocol (Illumina). Using the Illumina HiSeq 2500 platform, the mRNA libraries were sequenced with a per-sample target of 40–50 million 100-bp paired-end reads. The data were processed using Mount Sinai’s standard mapping pipeline [34] (using STAR, 35 Picard [36], and Subread [37]). RNA-SeQC [38] was used to perform quality control on the mapped data.

### Bronchoalveolar lavage (BAL) sample collection and profiling

We collected bronchial samples from 44 children with asthma in the cohort who underwent bronchoscopy for clinical care. BAL was performed in the right middle lobe using three successive aliquots of sterile saline (1 mL/kg body weight, up to a maximum of 30 mL). BAL fluid samples were processed for cell counts using cytospin preparations stained with hematoxylin and eosin (H&E). Bronchial and nasal samples were collected at the same time from these participants.

### Identification of air pollutant-associated transcripts

Differential expression analysis was performed on the nasal transcriptome data using DESeq2 [39] to identify nasal transcripts associated with ozone and/or PM_2.5_. Ozone-associated nasal transcripts were identified with adjustment for the levels of other major air pollutants (PM_2.5_, CO, NO_2_, SO_2_, and PMc). Similarly, PM_2.5_-associated nasal transcripts were identified with adjustment for the levels of other major air pollutants (ozone, CO, NO_2_, SO_2_, and PMc). Age, sex, and race/ethnicity were included as covariates for the models. Air pollutant levels were normalized using min–max normalization. Specifically, each pollutant was scaled to a 0–1 range using the rescale() function from the scales R package, preserving the relative distribution of values while standardizing their scale for downstream analyses. We adjusted for multiple testing by a permutation-based approach with 10,000 iterations to calculate false discovery rate (FDR) values [40, 41].

### Probabilistic causal (Bayesian) networks and key driver analysis

We built Bayesian networks, directed networks in which the edges are defined by conditional probabilities, 42–49 separately for participants with and without asthma using RIMBANet [50], a software package for reconstructing integrative molecular Bayesian networks. We mapped expression quantitative trait loci (eQTLs) to use as priors for Bayesian network construction. To map eQTLs, we used methods described in our prior studies [51, 52]. For variant calling on RNA-seq data, RNA-seq reads were aligned to the reference genome (GRCh38) using STAR aligner,^35^ and variants were called using the “HaplotypeCaller” GATK [53]. BEAGLE 5.1 [54] was used for genotype phasing and imputation against the 1000 Genomes reference haplotypes for GRCh38 [55]. We hard-called the variants using PLINK2 [56] after filtering out variants with low imputation accuracy (dosage R-squared < 0.8) and low minor allele frequency (MAF < 0.01). The eQTLs were identified where cis SNPs (within 1 Mb of the transcription start or end site of the gene) were associated with transcript expression at FDR ≤ 0.05 using Matrix eQTL. [57]

To construct the Bayesian networks using RIMBANet [50], the nasal RNA-seq data were normalized by using the R package edge R [58] after filtering out lowly expressed transcripts by retaining transcripts where counts per million were greater than 1 in at least one-quarter of the total number of samples. Transcripts with cis-eQTLs were used as genetic priors to infer causal directions between transcripts in the normalized nasal RNA-seq data. Monte Carlo Markov Chain (MCMC) simulation was used to identify 1,000 potentially plausible networks, which were then combined into a consensus network. Edges that appeared in more than 30% of the 1,000 networks were used to define the final consensus network.

Key driver analysis using the KDA R package [59] was then performed to identify the key drivers within the respective constructed Bayesian networks for participants with and without asthma. Within a Bayesian network, nodes that are more connected and located upstream will have more substantial impact on downstream genes. Such nodes are considered key drivers. Key driver analysis mathematically identifies causal modulators of the regulatory state of functionally relevant gene groups [59]. Key drivers have been found to be reproducible and successfully validated in different contexts [45–49]. Subnetworks including transcripts within a path length of 7 from ozone- and PM_2.5_-associated transcripts in the Bayesian networks were used as background networks for the key driver analyses.

### Gene ontology analysis

We used PANTHER [60] for Gene Ontology (GO) analysis. Fisher’s Exact was used to calculate the significance, and the significance threshold was fold enrichment ≥ 1 and FDR ≤ 0.05. The top GO terms in each category based on hierarchical sorting are shown.

Correlation analyses between nasal transcripts, bronchial transcripts, and BAL cellular counts.

For the 44 children with asthma for whom we had paired nasal and bronchial samples (Additional File 1: Table S1), Spearman correlation was performed between the expression levels of nasal transcripts in the key driver networks and respective bronchial transcripts. P-values were adjusted for multiple testing using a permutation-based approach with 10,000 iterations to calculate false discovery rate (FDR) values with a threshold FDR ≤ 0.05 used to identify significant results [40, 41]. The same approach was used to test for correlations between bronchial transcript expression and BAL cellular counts.

## Results

### Characteristics of the study cohort

**Fig. 2:**
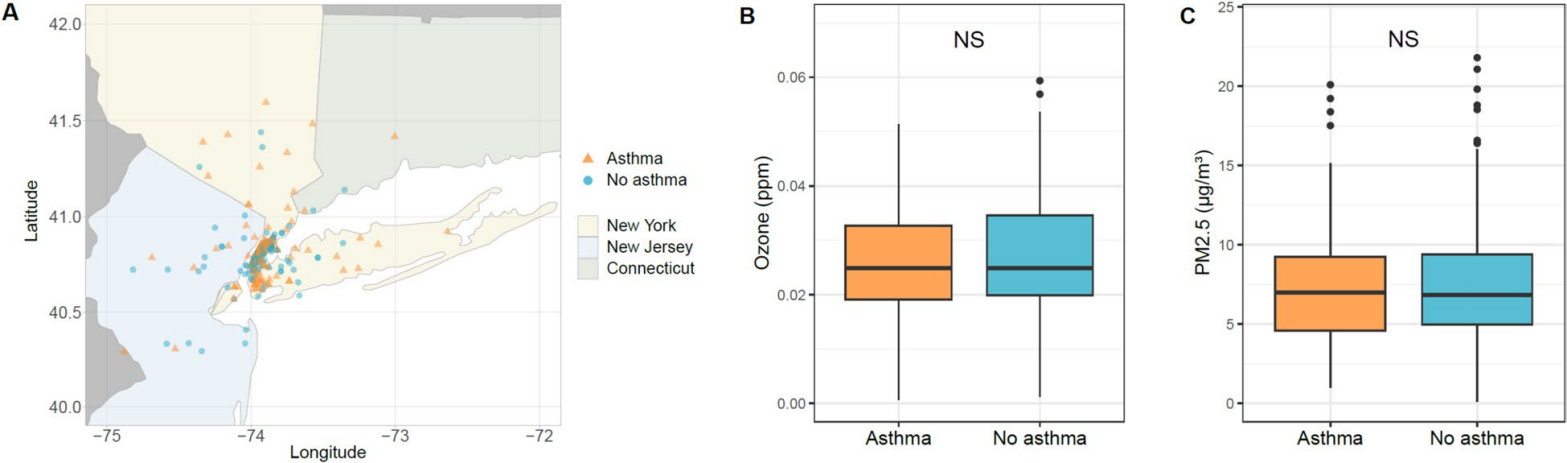
Home locations and pollutant levels for the participants. **A** Participants’ homes were located in the New York tri-state metropolitan area including New York, New Jersey, and Connecticut. Points indicate the locations of participant homes at the time of sampling. **B** Ozone levels at home locations on the date of sample collection. **C** PM_2.5_ levels at home locations on the date of sample collection. (NS = Not Significant)

The study cohort of 307 children (median age 13.3 years) was recruited from the Mount Sinai Health System, New York, NY, USA and included 167 participants with asthma and 140 subjects without asthma (Table 1). Participants with asthma had physician-diagnosed mild to severe persistent asthma based on the National Asthma Education and Prevention Program (NAEPP) Expert Panel Report 3 (EPR3) guidelines [32] and demonstrated airway hyperresponsiveness by pre- and post-bronchodilator spirometry or methacholine challenge, with the exception of 4 children age 4–6 years with reactive airway symptoms who did not successfully complete spirometry meeting American Thoracic Society guidelines for quality control [61] but were assigned to the asthma group based on the clinical assessment of pediatric pulmonologists following NAEPP EPR3 guidelines [32]. Participants without asthma had no personal or family history of asthma in first degree relatives and also demonstrated normal pre- and post-bronchodilator spirometry. Consistent with the population of our health system, the participants’ homes were in the tri-state area of New York, New Jersey, and Connecticut (Fig. 2A). The participants with asthma were younger and more likely to be male, Black, and Latino compared to their counterparts without asthma (Table 1). As anticipated, predicted forced expiratory volume in the first second (FEV1%) and the ratio of the forced expiratory volume in the first second to forced vital capacity (FEV1/FVC) by spirometry were significantly lower in the children with asthma. The median Asthma Control Test (ACT) score of the asthmatic participants was 18 (IQR = 6), while all the children without asthma uniformly scored the highest possible score (ACT = 25) as expected. Among the participants with asthma, 56 (34%) had very poorly controlled asthma (ACT 5–15), 63 (38%) were not well-controlled (ACT 16–19), and 45 (27%) were well-controlled (ACT 20–25) based on Global Initiative for Asthma categories [62]. All participants provided nasal samples, and we were also able to obtain bronchial samples from 44 participants with asthma who underwent clinically indicated bronchoscopy for severe asthma. Accordingly, the children who underwent bronchoscopy had lower ACT (mean 13.4, SD 4.3) and were more likely to be on higher step therapy compared to the overall cohort, although their pollutant exposures were comparable (Additional File 1: Table S1).

**Table 1:** Characteristics of the study cohort

	All (*n* = 307)	Asthma (*n* = 167)	No asthma (*n* = 140)	*P*-value
Age	13.3 (4.2)	12.4 (3.7)	14.4 (4.5)	3.7E-05
Sex (female)	151 (49%)	71 (43%)	80 (57%)	0.012
Race				2.5E-03
Asian	24 (8%)	5 (3%)	19 (14%)	
Black	59 (19%)	40 (24%)	19 (14%)	
White	146 (48%)	76 (46%)	70 (50%)	
Mixed race	40 (13%)	23 (14%)	17 (12%)	
Other	38 (12%)	23 (14%)	15 (11%)	
Ethnicity				
Latino	82 (27%)	56 (34%)	26 (19%)	4.2E-03
Season of study visit				0.57
Spring	80 (26%)	41 (25%)	39 (28%)	
Summer	82 (27%)	41 (25%)	41 (29%)	
Fall	71 (23%)	41 (25%)	30 (21%)	
Winter	74 (24%)	44 (26%)	30 (21%)	
Asthma Control Test (ACT) scores (mean, sd)	20.5 (5.2)	16.6 (4.3)	25 (0.0)	2.1E-58
FEV1% (mean, sd)	88.7 (14.8)	86.2 (17.5)	91.3 (10.7)	3.5E-03
FEV1/FVC (mean, sd)	83.6 (9.8)	79.3 (10.5)	88.1 (6.6)	2.6E-15
Allergic rhinitis	104 (34%)	72 (43%)	32 (23%)	2.6E-04
Asthma medication use, current				
Short-acting inhaled beta agonist (SABA)	39 (13%)	39 (23%)	0 (0%)	8.5E-12
Inhaled corticosteroid (ICS)	8 (3%)	8 (5%)	0 (0%)	8.8E-03
Combined inhaled corticosteroid (ICS)/LABA	29 (9%)	29 (17%)	0 (0%)	7.3E-09
Leukotriene receptor antagonist	23 (7%)	23 (14%)	0 (0%)	5.6E-07
Oral corticosteroid	5 (2%)	5 (3%)	0 (0%)	0.065
Omalizumab	3 (1%)	3 (2%)	0 (0%)	0.25
Pollutant levels				
Ozone (ppm)	0.03 (0.01)	0.03 (0.01)	0.03 (0.01)	0.37
PM_2.5_ (µg/m^3^)	7.38 (4.2)	7.07 (3.8)	7.78 (4.7)	0.19
CO (ppm)	0.31 (0.2)	0.30 (0.2)	0.34 (0.2)	0.043
SO_2_ (ppb)	0.63 (0.7)	0.65 (0.7)	0.61 (0.8)	0.66
NO_2_ (ppb)	18.63 (8.2)	18.42 (7.5)	18.88 (9.0)	0.65
PM_C_ (µg/m^3^)	6.81 (3.8)	6.44 (3.4)	7.20 (4.3)	0.13

Fisher Exact test for categorical variables, T-test for continuous variables. Mean (SD) or number (%) are shown

Ozone and PM_2.5_ levels on the date of nasal sampling at each participant’s home zip code were obtained from daily air quality data measured by the United States Environmental Protection Agency (EPA) [33]. Ozone (Fig. 2B) and PM_2.5_ (Fig. 2C) levels did not differ for participants with and without asthma. The levels of other commonly measured criteria air pollutants (SO_2_, NO_2_, and PMc (particles of aerodynamic diameter between 2.5 and < 10 μm)) also did not differ between the groups (Table 1) except for CO; all models in this study were adjusted for the levels of all criteria air pollutants.

### Ozone and PM_2.5_ networks in children with asthma are enriched for pro-inflammatory adaptive immune processes

To examine the effects of ozone and PM_2.5_ exposure on the airway transcriptome in asthma, we first built models with air pollutant and nasal transcriptome data from children with asthma. Nasal transcript level was the outcome and each pollutant level (ozone or PM_2.5_) was the main predictor with adjustment for age, sex, race/ethnicity, and the levels of all other major air pollutants shown in Table 1. Among the participants with asthma, we identified 262 nasal transcripts associated with ozone level (Additional File 1: Table S2) and 395 nasal transcripts associated with PM_2.5_ level (Additional File 1: Table S3) with β ≥ 1 and FDR ≤ 0.05. Nineteen transcripts overlapped between the ozone and PM_2.5_-associated transcripts.

**Fig. 3:**
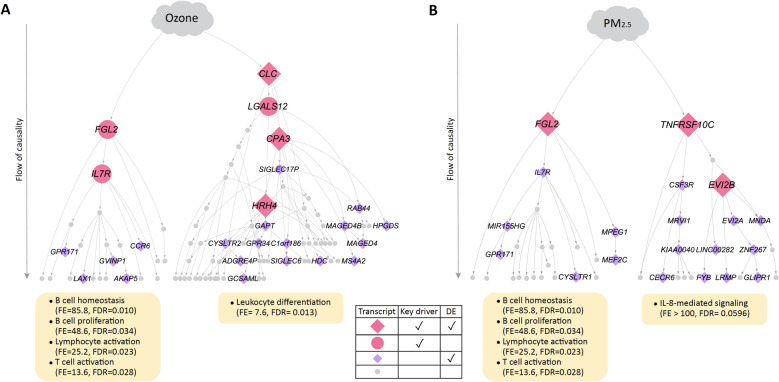
Probabilistic causal networks and key drivers for ozone and PM_2.5_ exposure in children with asthma. Probabilistic causal network for nasal transcripts associated with (**A**) ozone and (**B**) PM_2.5_ exposure in children with asthma. The causality flow is from top to bottom; transcripts at the highest levels have the greatest causal impact on the expression of transcripts in the network. Key drivers identified by the causal network construction and analyses are shown in pink. Transcripts shown are those within a path length of 7 of differentially expressed (DE) transcripts associated with ozone or PM_2.5_ level at beta ≥ 1 and FDR ≤ 0.05. Gene ontology (GO) terms for transcripts in the *FGL2* and *CLC* subnetworks are shown in yellow boxes based on fold enrichment (FE) ≥ 1 and FDR ≤ 0.05. The GO term shown for the *TNFRSF10C* subnetwork was the term with lowest FDR

We then built probabilistic causal (Bayesian) networks to characterize causal relationships among the ozone and PM_2.5_-associated nasal transcripts identified in these children with asthma. Bayesian networks are directed graphical models that represent a set of variables and their conditional dependencies where causal relationships between variables are represented [42–48, 50]. With these Bayesian networks, we then performed key driver analysis [59] to identify key driver transcripts (FDR ≤ 0.05) at the most upstream level of causal influence in the ozone and PM_2.5_ networks among these children with asthma. Transcripts at higher levels in these directed graphs exert greater causal impact on the networks for ozone- and PM_2.5_-associated transcripts shown (Fig. 3). Our analyses revealed six key drivers (*CLC*, *CPA3*, *FGL2*, *LGALS12, IL7R, HRH4*) for the ozone network (Fig. 3A) and three key driver transcripts (*TNFRSF10C, FGL2, EVI2B*) for the PM_2.5_ network (Fig. 3B). *FGL2* and *CLC* in the ozone network, and *FGL2* and *TNFRSF10C* in the PM_2.5_ network were at the most upstream level of causal influence in these respective networks. Sensitivity analyses where models were additionally adjusted for current medications demonstrated that the identified key drivers remained stable.

The *FGL2* key driver and its subnetwork were common to both the ozone and PM_2.5_ networks. Gene ontology (GO) enrichment analysis [60] of the *FGL2* subnetwork revealed enrichment for the biological processes of “B cell homeostasis” (fold enrichment = 85.8, FDR = 0.010), “B cell proliferation” (fold enrichment = 48.6, FDR = 0.034), “lymphocyte activation” (fold enrichment = 25.2, FDR = 0.023), and “T cell activation” (fold enrichment = 13.6, FDR = 0.028) (Fig. 3).

GO enrichment analyses of the subnetworks exclusive to the ozone and PM_2.5_ networks, respectively, revealed distinct processes, including “leukocyte differentiation” for the *CLC* subnetwork of the ozone network (fold enrichment = 7.6, FDR = 0.013) (Fig. 3A). While TNFRS10C was a statistically significant key driver for the PM_2.5_ network (FDR 1.1 × 10–4), GO enrichment analysis for its relatively small subnetwork identified “interleukin-8-mediated signaling” as the process with lowest FDR (fold enrichment > 100, FDR = 0.059) (Fig. 3B).

### The ozone network in children without asthma is enriched for DNA repair

**Fig. 4:**
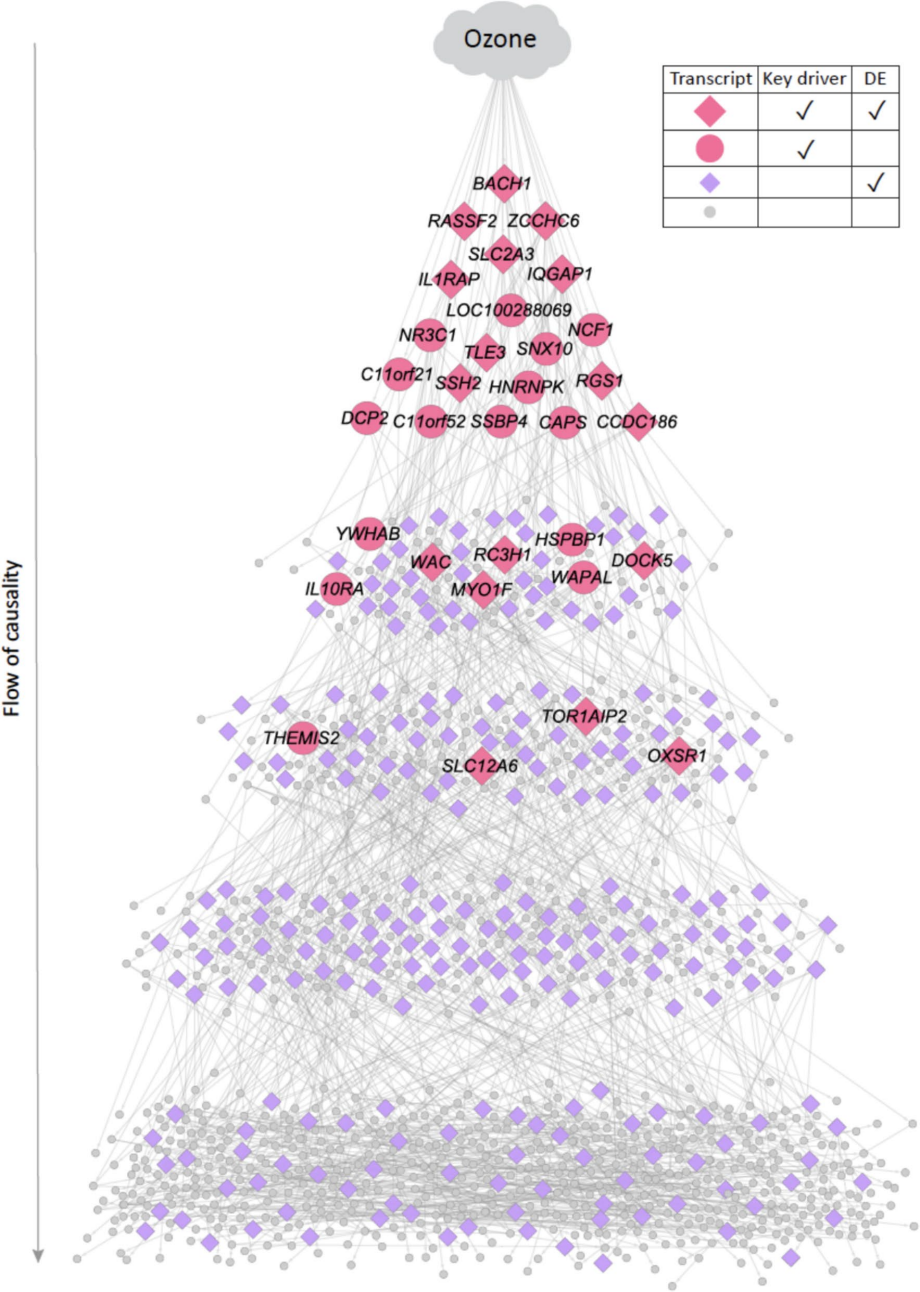
Probabilistic causal network and key drivers for ozone exposure in children without asthma. Probabilistic causal network for nasal transcripts associated with ozone exposure in children without asthma. The causality flow is from top to bottom; transcripts at the highest levels have the greatest causal impact on the expression of transcripts in the network. Key drivers identified by the causal network construction and analyses are shown in pink. Transcripts shown are those within a path length of 7 of differentially expressed (DE) transcripts associated with ozone level at beta ≥ 1 and FDR ≤ 0.05

In contrast to the findings in children with asthma, ozone and PM_2.5_ exposure were associated with nasal transcripts and key drivers that were markedly distinct in the children without asthma. These included 1,250 transcripts associated with ozone level (Additional File 1: Table S4) and 82 transcripts associated with PM_2.5_ level (Additional File 1: Table S5) at β ≥ 1 and FDR ≤ 0.05 among the children without asthma. Causal network construction revealed a highly connected network associated with ozone exposure in these non-asthmatic individuals, with key driver analysis revealing 32 key driver transcripts (Fig. 4), none of which overlapped with key drivers of the ozone network in children with asthma (Fig. 3A). Among these 32 key drivers, 20 were at the most upstream level of causal influence (Fig. 4). The ozone key drivers and the downstream transcripts in the participants without asthma were most significantly enriched for “DNA repair” (fold enrichment = 2.05, FDR = 3.67E-04) and “inflammatory response” (fold enrichment = 2.04, FDR = 2.17E-04). In the children without asthma, there were relatively few transcripts associated with PM_2.5_ exposure and only one key driver (*CST1*) of the PM_2.5_ network.

### Positive correlation between nasal and bronchial expression of ozone and PM_2.5_ network transcripts in children with asthma

**Fig. 5:**
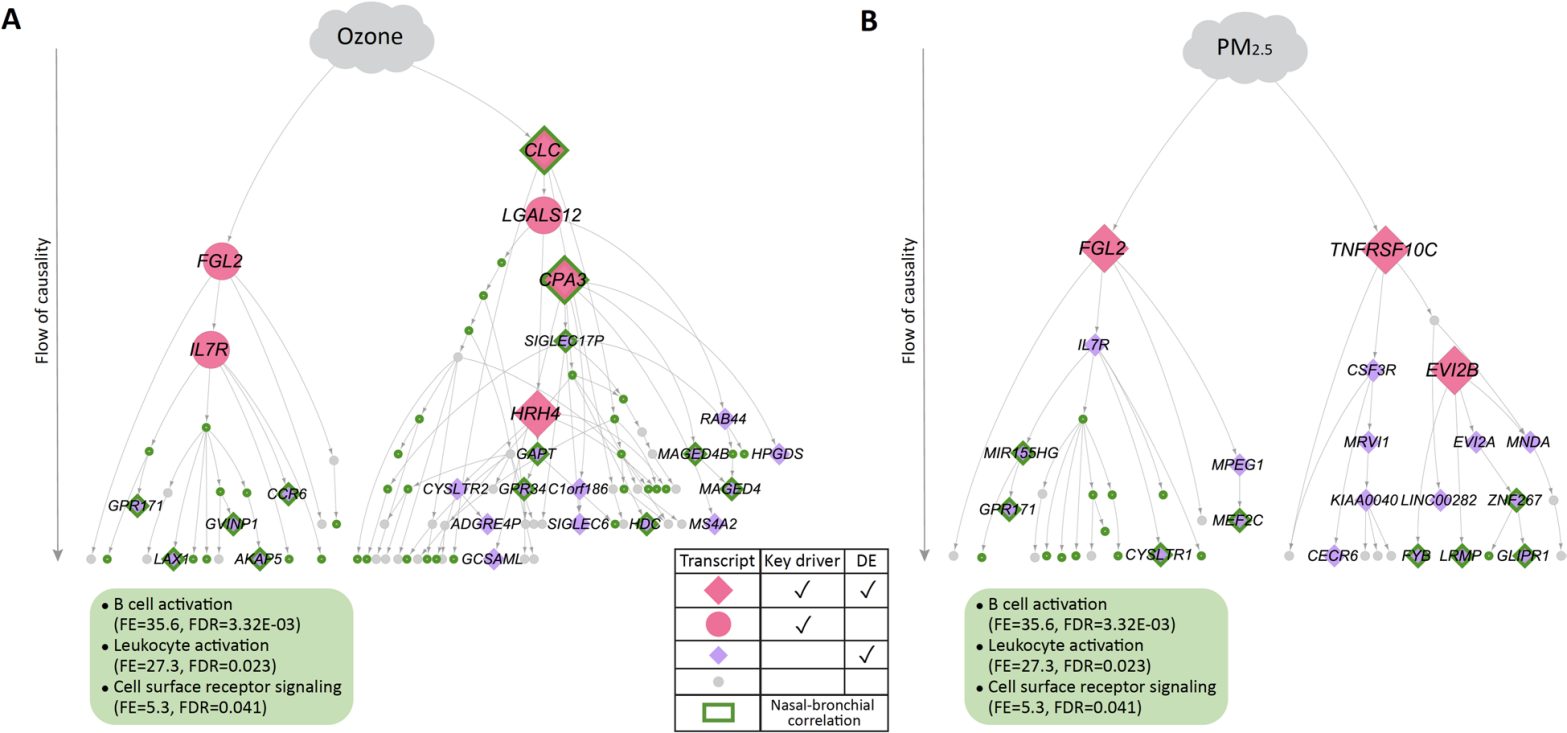
Correlation between nasal and bronchial expression of many transcripts in the ozone and PM_2.5_ networks for children with asthma. Transcripts with green borders indicate significant correlations (Spearman correlation FDR ≤ 0.05) between nasal and bronchial expression of transcripts in the (**A**) ozone and (**B**) PM_2.5_ networks for children with asthma from Fig. 3. All significant correlations identified were positive. GO terms for transcripts in the *FGL2* subnetwork with correlated nasal and bronchial expression are shown in the green boxes. There were no significant GO terms for correlated nasal and bronchial transcripts in the *CLC* and *TNFRSF10C* subnetworks

To investigate the degree to which nasal expression of ozone and PM_2.5_ key drivers is associated with their expression in the lower airway, we next tested for correlation between nasal and bronchial expression of the transcripts shown in Fig. 3 in the 44 participants with asthma for whom we had paired nasal and bronchial transcriptome profiles (Additional File 1: Table S1). Nasal expression of 53% of transcripts in the ozone network (Fig. 5A) and 45% of transcripts in the PM_2.5_ network (Fig. 5B) significantly correlated (Spearman correlation FDR ≤ 0.05) with bronchial expression of the respective transcripts, with all correlations positive (r = 0.3—0.7). Within the *FGL2* subnetwork, which was found in both the ozone and PM_2.5_ networks of participants with asthma, there was correlation between nasal and bronchial transcript expression for 65% of subnetwork transcripts. These nasal-bronchial correlated transcripts in the *FGL2* subnetwork were enriched for B cell activation, leukocyte activation, and cell surface receptor signaling (Fig. 5).

### Correlation between bronchial expression of ozone/PM_2.5_ key drivers and BAL cell counts

**Fig. 6:**
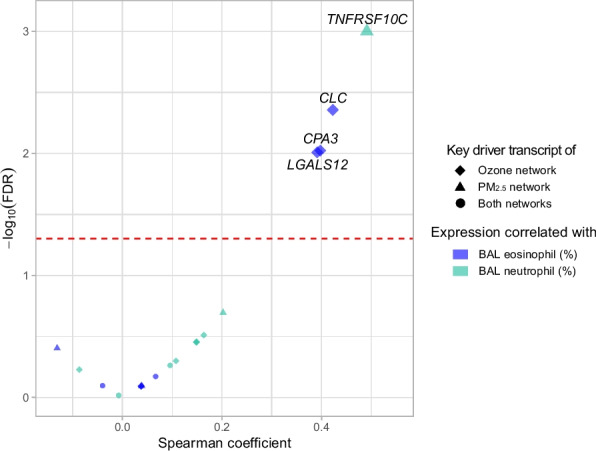
Correlation between bronchial expression of ozone/PM_2.5_ key drivers and BAL cell counts. Spearman correlations between bronchial expression of ozone/PM_2.5_ key drivers and bronchoalveolar lavage (BAL) eosinophil and neutrophil abundances are shown. The red line indicates FDR = 0.05

As many of the identified key drivers in participants with asthma (Fig. 3) were eosinophil marker transcripts (e.g. *CLC*) and transcripts associated with eosinophil activity (e.g. *CPA3*), we next tested for correlation between key driver transcript expression and eosinophil and neutrophil abundances in bronchoalveolar lavage (BAL) fluid from participants with asthma. BAL fluid samples had a mean eosinophil level of 2.9% (SD, 5.8%) and mean neutrophil level of 15.8% (SD, 24.8%). While nasal expression of the key drivers shown in Fig. 3 was not associated with BAL cell counts, bronchial transcript expression of these key drivers significantly correlated with BAL eosinophil and neutrophil abundances as shown in Fig. 6. Specifically, bronchial expression of ozone key drivers *CLC* (Spearman coefficient r = 0.42, FDR = 0.0044), *CPA3* (r = 0.40, FDR = 0.0095), and *LGALS12* (r = 0.39, FDR = 0.0098) positively correlated with BAL eosinophil abundance (Fig. 6). Additionally, bronchial expression of the PM_2.5_ key driver *TNFRSF10C* positively correlated with BAL neutrophil abundance (r = 0.49, FDR = 0.0010) (Fig. 6).

## Discussion

In this study, we found that children with and without asthma harbor very distinct airway transcriptome networks associated with ozone and PM_2.5_ exposure. Among asthmatic participants, these networks and their key drivers represented adaptive immune processes (Fig. 3), while in children without asthma, the airway transcriptome networks and their key drivers centered on DNA repair (Fig. 4). Our findings support that mechanisms of ozone and PM_2.5_ effects on the airway transcriptome differ in individuals with and without asthma, suggesting potential pathways for the disproportionate burden of air pollution on asthmatics. This study expands the field by revealing causal relationships within airway transcriptome subnetworks associated with ozone and PM_2.5_ exposure and identifying key drivers exerting greatest causal influence on the distinct processes found for children with and without asthma. Further, we found that transcript expression of the identified key drivers in the nasal airway correlated with their expression in the bronchial airway (Fig. 5) as well as with bronchial cellular abundances (Fig. 6), supporting the impact of these causal networks and key drivers on the pathobiology of asthma. Few studies of asthma in children have paired findings from the upper and lower airways given the challenges of bronchial sampling.

Our study contributes new findings to a large body work on relationships between PM_2.5_, ozone, and pro-inflammatory processes in airway epithelium and in asthma [1–23, 63]. For example, investigators previously found that exposure to PM_2.5_ organic extract activates a mucus secretory expression program in cultured nasal epithelial cells [11]. In urban US children with asthma, regional PM_2.5_ was found to be associated with nasal blow and nasal lavage modules representing epithelial cell processes within undirected correlational networks, and ozone was associated with modules representing Type 2 inflammation [12]. This study’s causal networks, key driver analyses, and inclusion of both children with and without asthma for direct juxtaposition revealed that ozone and PM_2.5_ are each associated with specific subnetworks of upper airway transcript expression, where transcripts relate to one another in a directed fashion, and with these subnetworks markedly distinct between participants with (Fig. 3) and without (Fig. 4) asthma.

In children with asthma, we found an *FGL2*-driven subnetwork for B cell proliferation and T cell activation associated with both PM_2.5_ and ozone exposure; a *CLC*- and *CPA3*-driven subnetwork for leukocyte differentiation associated with ozone; and a *TNFRSF10C*-driven subnetwork associated with PM_2.5_ (Fig. 3). A member of the fibrinogen family and key driver of both the ozone and PM_2.5_ networks in our study, *FGL2* (fibrinogen-like protein 2) blocks the Type 1 cytokines IL-2 and IFN-γ and activates the Type 2 cytokines IL-4 and IL-10 [64, 65]. Key drivers unique to the ozone network, *CLC* and *CPA3*, shape central effector cells in allergic inflammation, with *CLC* encoding Charcot-Leyden crystal protein/galectin-10 that serves as a carrier for the sequestration and vesicular transport of eosinophil granule cationic RNAses [66], and *CPA3* encoding carboxypeptidase 3, a mast cell-restricted protease released upon mast cell activation whose levels have been associated with eosinophilic airway inflammation [67, 68]. The key driver unique to the PM_2.5_ network, *TNFRSF10C* (TNF Receptor Superfamily Member 10c), is a member of the TNF receptor superfamily that promotes cellular differentiation, survival, and cytokine and chemokine production [69], and its expression in peripheral blood has been associated with treatment-resistant childhood asthma. [70]

Notably, in children without asthma, the causal network and key drivers associated with ozone were markedly distinct (Fig. 4), reflecting DNA repair and elements of immune regulation rather than the more consistently pro-inflammatory immune processes in participants with asthma (Fig. 3). Ozone is a strong oxidant that induces oxidative damage to cells and airways [71] and damages DNA [72]. A top key driver transcript of the ozone network in non-asthmatics, *BACH1* (BTB Domain and CNC Homolog 1) (Fig. 4) encodes a protein that represses oxidative stress, including in airway epithelial cells [73–75]. Consistent with the fact that air pollutant exposure is an insult to the airway for non-asthmatics as well, some key drivers of the ozone network in non-asthmatics are involved in airway inflammation (e.g. IL1RAP [76]). However, and notably, many of the key drivers in this network for non-asthmatics exert immune moderating effects (Fig. 4). For example, *ZCCHC6*, expressed in alveolar macrophages, prevents excessive inflammation by reducing IL-6, CXCL1, and CXCL5 secretion and neutrophil chemotaxis [77]. *IQGAP1* encodes the scaffolding protein IQ motif-containing GTPase-activating protein 1, whose binding to OX40L limits OX40 co-signaling, restricting inflammation by antigen-activated T cells [78]. *RASSF2* encodes a protein that suppresses NF-ҡB luciferase activity and diminishes levels of NF-ҡB/p50 [79]. Together, these findings suggest an airway transcriptional program in ozone-exposed non-asthmatics that includes protection from oxidative stress and moderation of inflammation.

Interestingly, in individuals without asthma, PM_2.5_ exposure was associated with few nasal transcripts, in contrast to PM_2.5_’s associations with many transcripts reflecting adaptive immune processes in children with asthma (Fig. 3). We surmise that this difference may be because healthy individuals can better clear PM_2.5_ from their airways compared to their asthmatic counterparts. Individuals with asthma have dysfunctional mucociliary clearance [80] that impairs removal of particulate matter including PM_2.5_, thus allowing such particulates to remain and induce inflammation. The protein encoded by the key driver of the PM_2.5_ network in children without asthma, CST1, is known to have protective roles, including the inhibition of allergen protease activity, [81] reduction of airway tight junction disruption [82], and association with successful symptom control and decreased Type 2 cells in a clinical trial of immunotherapy for allergic rhinitis. [83]

Recognizing the many detrimental effects of PM_2.5_ exposure, the United States EPA lowered the limit of primary annual PM_2.5_ emission standard from 12.0 µg/m^3^ to 9.0 µg/m^3^ in May 2024 [84, 85]. However, the EPA’s limit for ground-level ozone of 0.070 ppm has not changed since 2015 [86, 87], and the EPA stated in 2020 that there was little new information to suggest need for revision [88]. Although the level of ozone exposure in our study cohort was below this limit as shown in Fig. 2B, these below-limit ozone levels were still significantly associated with airway inflammatory processes in children with asthma (Fig. 3). The clinical impact of current levels of ozone exposure below the EPA limit and the attendant inflammatory processes we detected in this study are supported by others’ findings that below-limit ozone exposure is associated with increased short-acting beta-2 agonist use by individuals with asthma. [89]

We appreciate the limitations of this study. We studied an urban population from the New York metropolitan area that does not include exposures or outcomes that individuals living in rural or other geographic areas might experience. We used the EPA’s outdoor air quality measurements for ozone and PM_2.5_ at each participant’s home zip code [90] rather than personal monitors for exposure assessment. As transcriptomics has the capacity to reflect molecular responses to acute exposures, 90, 91 we focused on short-term rather than long-term air pollutant exposure but do recognize that chronic exposure can also exert effects. Last, although we could not obtain bronchial samples for every participant in the cohort because bronchoscopy is an invasive procedure not indicated for healthy individuals, and it is infrequently performed in children with asthma, our health system’s severe asthma program where clinically indicated bronchoscopies are conducted enabled us to collect bronchial samples from a substantial subset of the cohort. Our sample of asthmatic children with paired upper and lower airway samples is large compared to prior work and powered our findings of significant correlation between the expression of nasal key drivers, bronchial transcript expression, and bronchial cell counts (Figs. 5 and 6).

## Conclusions

In summary, in this study of air pollutants, upper and lower airway transcriptomes, and bronchial cellular counts, we identified key driver transcripts associated with ozone and PM_2.5_ exposure that causally influence downstream transcriptional networks that are markedly distinct between children with and without asthma. Our results suggest that air pollutant effects on the airway may differ in children with and without asthma. The key drivers identified represent potential high-yield targets for intervention and therapy of pollution-exacerbated asthma. These findings broaden our understanding of why individuals with asthma are more susceptible to air pollution and provide motivation for policies to further reduce ozone and PM_2.5_ in our environment.

## Supplementary Information

Additional file 1. Supplementary Tables. Table S1. Characteristics of the children with bronchial samples. Table S2. Nasal transcripts associated with ozone level in children with asthma. Table S3. Nasal transcripts associated with PM2.5 level in children with asthma. Table S4. Nasal transcripts associated with ozone level in children without asthma. Table S5. Nasal transcripts associated with PM2.5 level in children without asthma.

## Data Availability

Data for this study (Accession number syn63710705) are available at Synapse (https://www.synapse.org), a software platform for open, reproducible data-driven science. *Materials:* This study did not generate new materials or unique reagents.

## Abbreviations

ACT: Asthma control test
BACH1: BTB domain and CNC homolog 1
BAL: Bronchoalveolar lavage
CO: Carbon monoxide
CLC: Charcot-Leyden crystal
CPA3: Carboxypeptidase 3
CST1: Cystatin 1
CXCL: Chemokine C-X-C motif ligand
EPA: Environmental protection agency
EVI2B: Ecotropic viral integration site 2B
FDR: False discovery rate
FEV1%: Percent predicted forced expiratory volume in the first second
FGL2: Fibrinogen-like protein 2
FVC: Forced vital capacity
GO: Gene ontology
HRH4: Histamine receptor H4
ICS: Inhaled corticosteroid
IL: Interleukin
IL1RAP: Interleukin 1 receptor accessory protein
IL7R: Interleukin 7 receptor
IQGAP1: IQ motif-containing GTPase-activating protein 1
LABA: Long-acting beta agonist
LGALS12: Galectin 12
NAEPP EPR3: National asthma education and prevention program expert panel report 3
NF-ҡB: Nuclear factor kappa B
NO2: Nitrogen dioxide
OX40L: OX40 ligand
PM2.5: Particulate matter ≤ 2.5 µm
PMc: Particulate matter > 2.5 µm and ≤ 10 µm
ppm: parts per million
RASSF2: Ras association domain family member 2
SABA: Short-acting beta agonist
SO2: Sulfur dioxide
TNFRSF10C: TNF receptor superfamily member 10c
ZCCHC6: Zinc finger CCHRC-domain containing protein 6

## References

[1] Agache I, Canelo-Aybar C, Annesi-Maesano I, Cecchi L, Rigau D, Rodriguez-Tanta LY, et al. The impact of outdoor pollution and extreme temperatures on asthma-related outcomes: a systematic review for the EAACI guidelines on environmental science for allergic diseases and asthma. Allergy. 2024;79:1725–60. 38311978 10.1111/all.16041

[2] Zanobetti A, Ryan PH, Coull BA, Luttmann-Gibson H, Datta S, Blossom J, et al. Early-life exposure to air pollution and childhood asthma cumulative incidence in the ECHO CREW consortium. JAMA Netw Open. 2024;7:e240535. 38416497 10.1001/jamanetworkopen.2024.0535 PMC10902721

[3] Pfeffer PE, Mudway IS, Grigg J. Air pollution and asthma: mechanisms of harm and considerations for clinical interventions. Chest. 2021;159:1346–55. 33461908 10.1016/j.chest.2020.10.053

[4] Zhang Y, Eckel SP, Berhane K, Garcia E, Muchmore P, Molshatzki NB, et al. Long-term exposures to air pollutants affect F (eNO) in children: a longitudinal study. Eur Respir J. 2021. 10.1183/13993003.00705-2021. 34503981 10.1183/13993003.00705-2021 PMC9618404

[5] Li X, Chen Q, Zheng X, Li Y, Han M, Liu T, et al. Effects of ambient ozone concentrations with different averaging times on asthma exacerbations: a meta-analysis. Sci Total Environ. 2019;691:549–61. 31325855 10.1016/j.scitotenv.2019.06.382

[6] Anenberg SC, Mohegh A, Goldberg DL, Kerr GH, Brauer M, Burkart K, et al. Long-term trends in urban NO(2) concentrations and associated paediatric asthma incidence: estimates from global datasets. Lancet Planet Health. 2022;6:e49–58. 34998460 10.1016/S2542-5196(21)00255-2

[7] Liu W, Cai M, Long Z, Tong X, Li Y, Wang L, et al. Association between ambient sulfur dioxide pollution and asthma mortality: evidence from a nationwide analysis in China. Ecotoxicol Environ Saf. 2023;249:114442. 38321661 10.1016/j.ecoenv.2022.114442

[8] Cisneros R, Gharibi H, Entwistle MR, Tavallali P, Singhal M, Schweizer D. Nitrogen dioxide and asthma emergency department visits in California, USA during cold season (November to February) of 2005 to 2015: a time-stratified case-crossover analysis. Sci Total Environ. 2021;754:142089. 33254941 10.1016/j.scitotenv.2020.142089

[9] Olaniyan T, Jeebhay M, Roosli M, Naidoo RN, Kunzli N, de Hoogh K, et al. The association between ambient NO(2) and PM(2.5) with the respiratory health of school children residing in informal settlements: a prospective cohort study. Environ Res. 2020;186:109606. 32371276 10.1016/j.envres.2020.109606

[10] Zhao Y, Hu J, Tan Z, Liu T, Zeng W, Li X, et al. Ambient carbon monoxide and increased risk of daily hospital outpatient visits for respiratory diseases in Dongguan, China. Sci Total Environ. 2019;668:254–60. 30852202 10.1016/j.scitotenv.2019.02.333

[11] Montgomery MT, Sajuthi SP, Cho SH, Everman JL, Rios CL, Goldfarbmuren KC, et al. Genome-wide analysis reveals mucociliary remodeling of the nasal airway epithelium induced by urban PM(2.5). Am J Respir Cell Mol Biol. 2020;63:172–84. 32275839 10.1165/rcmb.2019-0454OC PMC7397762

[12] Altman MC, Kattan M, O’Connor GT, Murphy RC, Whalen E, LeBeau P, et al. Associations between outdoor air pollutants and non-viral asthma exacerbations and airway inflammatory responses in children and adolescents living in urban areas in the USA: a retrospective secondary analysis. Lancet Planet Health. 2023;7:e33–44. 36608946 10.1016/S2542-5196(22)00302-3 PMC9984226

[13] Liao J, Gheissari R, Thomas DC, Gilliland FD, Lurmann F, Islam KT, et al. Transcriptomic and metabolomic associations with exposures to air pollutants among young adults with childhood asthma history. Environ Pollut. 2022;299:118903. 35091019 10.1016/j.envpol.2022.118903 PMC8925195

[14] Cheng H, Narzo AD, Howell D, Yevdokimova K, Zhang J, Zhang X, et al. Ambient air pollutants and traffic factors were associated with blood and urine biomarkers and asthma risk. Environ Sci Technol. 2022;56:7298–307. 35239329 10.1021/acs.est.1c06916

[15] Gref A, Merid SK, Gruzieva O, Ballereau S, Becker A, Bellander T, et al. Genome-wide interaction analysis of air pollution exposure and childhood asthma with functional follow-up. Am J Respir Crit Care Med. 2017;195:1373–83. 27901618 10.1164/rccm.201605-1026OC PMC5443897

[16] Enweasor C, Flayer CH, Haczku A. Ozone-induced oxidative stress, neutrophilic airway inflammation, and glucocorticoid resistance in asthma. Front Immunol. 2021;12:631092. 33717165 10.3389/fimmu.2021.631092 PMC7952990

[17] Nassikas N, Spangler K, Fann N, Nolte CG, Dolwick P, Spero TL, et al. Ozone-related asthma emergency department visits in the US in a warming climate. Environ Res. 2020;183:109206. 32035409 10.1016/j.envres.2020.109206 PMC7167359

[18] Wei Y, Qiu X, Sabath MB, Yazdi MD, Yin K, Li L, et al. Air pollutants and asthma hospitalization in the Medicaid population. Am J Respir Crit Care Med. 2022;205:1075–83. 35073244 10.1164/rccm.202107-1596OC PMC9851478

[19] Liu Y, Pan J, Zhang H, Shi C, Li G, Peng Z, et al. Short-term exposure to ambient air pollution and asthma mortality. Am J Respir Crit Care Med. 2019;200:24–32. 30871339 10.1164/rccm.201810-1823OC

[20] Rosenquist NA, Metcalf WJ, Ryu SY, Rutledge A, Coppes MJ, Grzymski JJ, et al. Acute associations between PM(2.5) and ozone concentrations and asthma exacerbations among patients with and without allergic comorbidities. J Expo Sci Environ Epidemiol. 2020;30:795–804. 32094459 10.1038/s41370-020-0213-7

[21] Di Q, Dai L, Wang Y, Zanobetti A, Choirat C, Schwartz JD, et al. Association of short-term exposure to air pollution with mortality in older adults. JAMA. 2017;318:2446–56. 29279932 10.1001/jama.2017.17923 PMC5783186

[22] U.S. EPA. Integrated Science Assessment (ISA) for Ozone and Related Photochemical Oxidants (Final Report, Apr 2020). U.S. Environmental Protection Agency, Washington DC, EPA/600/R-20/012 2020.

[23] U.S. EPA. Integrated Science Assessment for Particulate Matter (Final Report, Dec 2019). U.S. Environmental Protection Agency, Washington DC, EPA/600/R-19/188 2019. 36630543

[24] Xu R, Yu P, Abramson MJ, Johnston FH, Samet JM, Bell ML, et al. Wildfires, global climate change, and human health. N Engl J Med. 2020;383:2173–81. 33034960 10.1056/NEJMsr2028985

[25] Xu L, Crounse JD, Vasquez KT, Allen H, Wennberg PO, Bourgeois I, et al. Ozone chemistry in western U.S. wildfire plumes. Sci Adv 2021; 7:eabl3648. 10.1126/sciadv.abl3648 PMC8654285 34878847

[26] Jin X, Fiore AM, Cohen RC. Space-based observations of ozone precursors within California wildfire plumes and the impacts on Ozone-NO(x)-VOC chemistry. Environ Sci Technol. 2023;57:14648–60. 37703172 10.1021/acs.est.3c04411

[27] Chen G, Guo Y, Yue X, Tong S, Gasparrini A, Bell ML, et al. Mortality risk attributable to wildfire-related PM(2.5) pollution: a global time series study in 749 locations. Lancet Planet Health. 2021;5:e579–87. 34508679 10.1016/S2542-5196(21)00200-X

[28] Neumann JE, Amend M, Anenberg S, Kinney PL, Sarofim M, Martinich J, et al. Estimating PM2.5-related premature mortality and morbidity associated with future wildfire emissions in the western US. Environ Res Lett. 2021. 10.1088/1748-9326/abe82b. 33868453 10.1088/1748-9326/abe82b PMC8048092

[29] Aguilera R, Corringham T, Gershunov A, Benmarhnia T. Wildfire smoke impacts respiratory health more than fine particles from other sources: observational evidence from Southern California. Nat Commun. 2021;12:1493. 33674571 10.1038/s41467-021-21708-0 PMC7935892

[30] Burke M, Childs ML, de la Cuesta B, Qiu M, Li J, Gould CF, et al. The contribution of wildfire to PM(2.5) trends in the USA. Nature. 2023;622:761–6. 37730996 10.1038/s41586-023-06522-6

[31] Burke M, Driscoll A, Heft-Neal S, Xue J, Burney J, Wara M. The changing risk and burden of wildfire in the United States. Proc Natl Acad Sci U S A. 2021. 10.1073/pnas.2011048118. 33431571 10.1073/pnas.2011048118 PMC7812759

[32] Services USDOHaH. Expert Panel Report 3: Guidelines for the Diagnosis and Management of Asthma - Full Report 2007; 2007.

[33] Air Data: Air Quality Data Collected at Outdoor Monitors Across the US. Daily Summary Data. United States Environmental Protection Agency, available at: https://www.epa.gov/outdoor-air-quality-data. Accessed 5/1/2024.

[34] Fromer M, Roussos P, Sieberts SK, Johnson JS, Kavanagh DH, Perumal TM, et al. Gene expression elucidates functional impact of polygenic risk for schizophrenia. Nat Neurosci. 2016;19:1442–53. 27668389 10.1038/nn.4399 PMC5083142

[35] Dobin A, Davis CA, Schlesinger F, Drenkow J, Zaleski C, Jha S, et al. STAR: ultrafast universal RNA-seq aligner. Bioinformatics. 2013;29:15–21. 23104886 10.1093/bioinformatics/bts635 PMC3530905

[36] Broad Institute. Picard. Available at: http://broadinstitute.github.io/picard/. Accessed 5/1/2024.

[37] Liao Y, Smyth GK, Shi W. Featurecounts: an efficient general purpose program for assigning sequence reads to genomic features. Bioinformatics. 2014;30:923–30. 24227677 10.1093/bioinformatics/btt656

[38] DeLuca DS, Levin JZ, Sivachenko A, Fennell T, Nazaire MD, Williams C, et al. RNA-SeQC: RNA-seq metrics for quality control and process optimization. Bioinformatics. 2012;28:1530–2. 22539670 10.1093/bioinformatics/bts196 PMC3356847

[39] Love MI, Huber W, Anders S. Moderated estimation of fold change and dispersion for RNA-seq data with DESeq2. Genome Biol. 2014;15:550. 25516281 10.1186/s13059-014-0550-8 PMC4302049

[40] Schadt EE, Molony C, Chudin E, Hao K, Yang X, Lum PY, et al. Mapping the genetic architecture of gene expression in human liver. PLoS Biol. 2008;6:e107. 18462017 10.1371/journal.pbio.0060107 PMC2365981

[41] Jayavelu AK, Schnoder TM, Perner F, Herzog C, Meiler A, Krishnamoorthy G, et al. Splicing factor YBX1 mediates persistence of JAK2-mutated neoplasms. Nature. 2020;588:157–63. 33239784 10.1038/s41586-020-2968-3

[42] Zhu J, Wiener MC, Zhang C, Fridman A, Minch E, Lum PY, et al. Increasing the power to detect causal associations by combining genotypic and expression data in segregating populations. PLoS Comput Biol. 2007;3:e69. 17432931 10.1371/journal.pcbi.0030069 PMC1851982

[43] Yang X, Zhang B, Molony C, Chudin E, Hao K, Zhu J, et al. Systematic genetic and genomic analysis of cytochrome P450 enzyme activities in human liver. Genome Res. 2010;20:1020–36. 20538623 10.1101/gr.103341.109 PMC2909567

[44] Watson CT, Cohain AT, Griffin RS, Chun Y, Grishin A, Hacyznska H, et al. Integrative transcriptomic analysis reveals key drivers of acute peanut allergic reactions. Nat Commun. 2017;8:1943. 29203772 10.1038/s41467-017-02188-7 PMC5715016

[45] Zhang B, Gaiteri C, Bodea LG, Wang Z, McElwee J, Podtelezhnikov AA, et al. Integrated systems approach identifies genetic nodes and networks in late-onset Alzheimer’s disease. Cell. 2013;153:707–20. 23622250 10.1016/j.cell.2013.03.030 PMC3677161

[46] Peters LA, Perrigoue J, Mortha A, Iuga A, Song WM, Neiman EM, et al. A functional genomics predictive network model identifies regulators of inflammatory bowel disease. Nat Genet. 2017;49:1437–49. 28892060 10.1038/ng.3947 PMC5660607

[47] Beckmann ND, Lin WJ, Wang M, Cohain AT, Charney AW, Wang P, et al. Multiscale causal networks identify VGF as a key regulator of Alzheimer’s disease. Nat Commun. 2020;11:3942. 32770063 10.1038/s41467-020-17405-z PMC7414858

[48] Cohain A, Divaraniya AA, Zhu K, Scarpa JR, Kasarskis A, Zhu J, et al. Exploring the reproducibility of probabilistic causal molecular network models. Pac Symp Biocomput. 2017;22:120–31. 27896968 10.1142/9789813207813_0013 PMC5161348

[49] Cohain AT, Barrington WT, Jordan DM, Beckmann ND, Argmann CA, Houten SM, et al. An integrative multiomic network model links lipid metabolism to glucose regulation in coronary artery disease. Nat Commun. 2021;12:547. 33483510 10.1038/s41467-020-20750-8 PMC7822923

[50] Zhu J, Sova P, Xu Q, Dombek KM, Xu EY, Vu H, et al. Stitching together multiple data dimensions reveals interacting metabolomic and transcriptomic networks that modulate cell regulation. PLoS Biol. 2012;10:e1001301. 22509135 10.1371/journal.pbio.1001301 PMC3317911

[51] Zhang L, Chun Y, Irizar H, Arditi Z, Grishina G, Grishin A, et al. Integrated study of systemic and local airway transcriptomes in asthma reveals causal mediation of systemic effects by airway key drivers. Genome Med. 2023;15:71. 37730635 10.1186/s13073-023-01222-2 PMC10512627

[52] Irizar H, Chun Y, Hsu HL, Li Y, Zhang L, Arditi Z, et al. Multi-omic integration reveals alterations in nasal mucosal biology that mediate air toxic effects on allergic rhinitis. Allergy. 2024;79:3047–61. 38796780 10.1111/all.16174 PMC11560721

[53] Van der Auwera GA, O'Connor BD. Genomics in the cloud: using Docker, GATK, and WDL in Terra: O'Reilly Media; 2020.

[54] Browning SR, Browning BL. Rapid and accurate haplotype phasing and missing-data inference for whole-genome association studies by use of localized haplotype clustering. Am J Hum Genet. 2007;81:1084–97. 17924348 10.1086/521987 PMC2265661

[55] 1000 Genomes Project Consortium, Auton A, Brooks LD, Durbin RM, Garrison EP, Kang HM, et al. A global reference for human genetic variation. Nature 2015; 526:68–74. 10.1038/nature15393 PMC4750478 26432245

[56] Chang CC, Chow CC, Tellier LC, Vattikuti S, Purcell SM, Lee JJ. Second-generation PLINK: rising to the challenge of larger and richer datasets. Gigascience. 2015;4:7. 25722852 10.1186/s13742-015-0047-8 PMC4342193

[57] Shabalin AA. Matrix eQTL: ultra fast eQTL analysis via large matrix operations. Bioinformatics. 2012;28:1353–8. 22492648 10.1093/bioinformatics/bts163 PMC3348564

[58] Robinson MD, McCarthy DJ, Smyth GK. edgeR: a bioconductor package for differential expression analysis of digital gene expression data. Bioinformatics. 2010;26:139–40. 19910308 10.1093/bioinformatics/btp616 PMC2796818

[59] Zhang, B. & Zhu, J. Identification of key causal regulators in gene networks. Proc. World Congr. Eng. 2013;II:5–8.

[60] Thomas PD, Ebert D, Muruganujan A, Mushayahama T, Albou LP, Mi H. PANTHER: making genome-scale phylogenetics accessible to all. Protein Sci. 2022;31:8–22. 34717010 10.1002/pro.4218 PMC8740835

[61] Graham BL, Steenbruggen I, Miller MR, Barjaktarevic IZ, Cooper BG, Hall GL, et al. Standardization of spirometry 2019 update. An official American Thoracic Society and European Respiratory Society technical statement. Am J Respir Crit Care Med. 2019;200:e70–88. 31613151 10.1164/rccm.201908-1590ST PMC6794117

[62] Global Initiative for Asthma. Global Strategy for Asthma Management and Prevention. 2024:https://ginasthma.org/wp-content/uploads/2022/07/GINA-Main-Report--FINAL-22-07-01-WMS.pdf, accessed 7/14/2025.

[63] Gauderman WJ, Avol E, Gilliland F, Vora H, Thomas D, Berhane K, et al. The effect of air pollution on lung development from 10 to 18 years of age. N Engl J Med. 2004;351:1057–67. 15356303 10.1056/NEJMoa040610

[64] Joller N, Lozano E, Burkett PR, Patel B, Xiao S, Zhu C, et al. Treg cells expressing the coinhibitory molecule TIGIT selectively inhibit proinflammatory Th1 and Th17 cell responses. Immunity. 2014;40:569–81. 24745333 10.1016/j.immuni.2014.02.012 PMC4070748

[65] Hu J, Yan J, Rao G, Latha K, Overwijk WW, Heimberger AB, et al. The duality of Fgl2 - secreted immune checkpoint regulator versus membrane-associated procoagulant: therapeutic potential and implications. Int Rev Immunol. 2016;35:325–39. 25259408 10.3109/08830185.2014.956360 PMC5257246

[66] Grozdanovic MM, Doyle CB, Liu L, Maybruck BT, Kwatia MA, Thiyagarajan N, et al. Charcot-Leyden crystal protein/galectin-10 interacts with cationic ribonucleases and is required for eosinophil granulogenesis. J Allergy Clin Immunol. 2020;146(377–89):e10. 10.1016/j.jaci.2020.01.013 PMC7375938 31982451

[67] Pejler G. The emerging role of mast cell proteases in asthma. Eur Respir J. 2019. 10.1183/13993003.00685-2019. 31371445 10.1183/13993003.00685-2019

[68] Winter NA, Qin L, Gibson PG, McDonald VM, Baines KJ, Faulkner J, et al. Sputum mast cell/basophil gene expression relates to inflammatory and clinical features of severe asthma. J Allergy Clin Immunol. 2021;148:428–38. 33609626 10.1016/j.jaci.2021.01.033

[69] Croft M, Duan W, Choi H, Eun SY, Madireddi S, Mehta A. TNF superfamily in inflammatory disease: translating basic insights. Trends Immunol. 2012;33:144–52. 22169337 10.1016/j.it.2011.10.004 PMC3299395

[70] Persson H, Kwon AT, Ramilowski JA, Silberberg G, Soderhall C, Orsmark-Pietras C, et al. Transcriptome analysis of controlled and therapy-resistant childhood asthma reveals distinct gene expression profiles. J Allergy Clin Immunol. 2015;136:638–48. 25863981 10.1016/j.jaci.2015.02.026

[71] Zhang JJ, Wei Y, Fang Z. Ozone pollution: a major health hazard worldwide. Front Immunol. 2019;10:2518. 31736954 10.3389/fimmu.2019.02518 PMC6834528

[72] Wagner JR, Madugundu GS, Cadet J. Ozone-induced DNA damage: a Pandora’s box of oxidatively modified DNA bases. Chem Res Toxicol. 2021;34:80–90. 33417438 10.1021/acs.chemrestox.0c00342

[73] Hayes JD, Dinkova-Kostova AT, Tew KD. Oxidative stress in cancer. Cancer Cell. 2020;38:167–97. 32649885 10.1016/j.ccell.2020.06.001 PMC7439808

[74] Jia M, Li Q, Guo J, Shi W, Zhu L, Huang Y, et al. Deletion of BACH1 attenuates atherosclerosis by reducing endothelial inflammation. Circ Res. 2022;130:1038–55. 35196865 10.1161/CIRCRESAHA.121.319540

[75] Zhang X, Guo J, Wei X, Niu C, Jia M, Li Q, et al. Bach1: function, regulation, and involvement in disease. Oxid Med Cell Longev. 2018;2018:1347969. 30370001 10.1155/2018/1347969 PMC6189649

[76] Badi YE, Salcman B, Taylor A, Rana B, Kermani NZ, Riley JH, et al. IL1RAP expression and the enrichment of IL-33 activation signatures in severe neutrophilic asthma. Allergy. 2023;78:156–67. 35986608 10.1111/all.15487 PMC10086999

[77] Kozlowski E, Wasserman GA, Morgan M, O’Carroll D, Ramirez NP, Gummuluru S, et al. The RNA uridyltransferase Zcchc6 is expressed in macrophages and impacts innate immune responses. PLoS ONE. 2017;12:e0179797. 28665939 10.1371/journal.pone.0179797 PMC5493306

[78] Okuyama Y, Nagashima H, Ushio-Fukai M, Croft M, Ishii N, So T. IQGAP1 restrains T-cell cosignaling mediated by OX40. FASEB J. 2020;34:540–54. 31914585 10.1096/fj.201900879RR

[79] Imai T, Toyota M, Suzuki H, Akino K, Ogi K, Sogabe Y, et al. Epigenetic inactivation of RASSF2 in oral squamous cell carcinoma. Cancer Sci. 2008;99:958–66. 18294275 10.1111/j.1349-7006.2008.00769.x PMC11158976

[80] Jesenak M, Durdik P, Oppova D, Franova S, Diamant Z, Golebski K, et al. Dysfunctional mucociliary clearance in asthma and airway remodeling - new insights into an old topic. Respir Med. 2023;218:107372. 37516275 10.1016/j.rmed.2023.107372

[81] Yao L, Yuan X, Fu H, Guo Q, Wu Y, Xuan S, et al. Epithelium-derived cystatin SN inhibits house dust mite protease activity in allergic asthma. Allergy. 2023;78:1507–23. 37026502 10.1111/all.15739

[82] Fukuoka A, Matsushita K, Morikawa T, Adachi T, Yasuda K, Kiyonari H, et al. Human cystatin SN is an endogenous protease inhibitor that prevents allergic rhinitis. J Allergy Clin Immunol. 2019;143(1153–62):e12. 10.1016/j.jaci.2018.06.035 30012514

[83] Lou H, Huang Y, Ouyang Y, Zhang Y, Xi L, Chu X, et al. *Artemisia annua*-sublingual immunotherapy for seasonal allergic rhinitis: a randomized controlled trial. Allergy. 2020;75:2026–36. 32030780 10.1111/all.14218

[84] U.S. EPA. National Ambient Air Quality Standards (NAAQS) for PM. U.S. Environmental Protection Agency, available at: https://www.epa.gov/pm-pollution/national-ambient-air-quality-standards-naaqs-pm. Accessed 10/14/2024. 2024.

[85] U.S. EPA. Reconsideration of the National Ambient Air Quality Standards for Particulate Matter. U.S. Environmental Protection Agency, available at: https://www.federalregister.gov/documents/2024/03/06/2024-02637/reconsideration-of-the-national-ambient-air-quality-standards-for-particulate-matter. Accessed 10/14/2024. 2024.

[86] U.S. EPA. Ozone National Ambient Air Quality Standards (NAAQS). U.S. Environmental Protection Agency, avalable at: https://www.epa.gov/ground-level-ozone-pollution/ozone-national-ambient-air-quality-standards-naaqs. Accessed 10/14/2024. 2024.

[87] U.S. EPA. National Ambient Air Quality Standards for Ozone. U.S. Environmental Protection Agency, available at: https://www.federalregister.gov/documents/2015/10/26/2015-26594/national-ambient-air-quality-standards-for-ozone. Accessed 10/14/2024. 2015.

[88] U.S. EPA. EPA Initiates New Review of the Ozone National Ambient Air Quality Standards to Reflect the Latest Science. U.S. Environmental Protection Agency, available at: https://www.epa.gov/newsreleases/epa-initiates-new-review-ozone-national-ambient-air-quality-standards-reflect-latest. Accessed 10/14/2024. 2023.

[89] Pepper JR, Barrett MA, Su JG, Merchant R, Henderson K, Van Sickle D, et al. Geospatial-temporal analysis of the impact of ozone on asthma rescue inhaler use. Environ Int. 2020;136:105331. 31836258 10.1016/j.envint.2019.105331

[90] Beck NB, J.Q K, Luchtel DL, Altman LC, Orsborn MT, Kenney JS. Ozone Can Increase the Expression of Intercellular Adhesion Molecule-1 and the Synthesis of Cytokines by Human Nasal Epithelial Cells. Inhal Toxicol 1994; 6:345–57.

[91] Nichols BG, Woods JS, Luchtel DL, Corral J, Koenig JQ. Effects of ozone exposure on nuclear factor-kappaB activation and tumor necrosis factor-alpha expression in human nasal epithelial cells. Toxicol Sci. 2001;60:356–62. 11248148 10.1093/toxsci/60.2.356

